# Association between telomere length and V_H_ gene mutation status in chronic lymphocytic leukaemia: clinical and biological implications

**DOI:** 10.1038/sj.bjc.6600763

**Published:** 2003-02-18

**Authors:** M Hultdin, R Rosenquist, U Thunberg, G Tobin, K-F Norrback, A Johnson, C Sundström, G Roos

**Affiliations:** 1 Department of Medical Biosciences, Pathology, Umeå University, SE-90185 Umeå, Sweden; 2 Departments of Genetics and Pathology, Uppsala University, SE-751 85 Uppsala, Sweden; 3 Department of Oncology, Radiology and Clinical Immunology, Uppsala University, SE-751 85 Uppsala, Sweden

**Keywords:** telomere length, immunoglobulin gene, somatic hypermutation, chronic lymphocytic leukaemia, prognosis

## Abstract

The immunoglobulin V_H_ gene mutation status can divide B-cell chronic lymphocytic leukaemia (CLL) into two entities with a different clinical course. Cases with unmutated V_H_ genes, considered to evolve from pregerminal centre (GC) cells, have a worse outcome compared to cases showing mutated V_H_ genes, that is, post-GC derived. Also, telomere length has been reported to be of prognostic significance in CLL. Interestingly, telomerase becomes activated during the GC reaction and an elongation of the telomeres occurs in GC B cells. We performed telomere length and V_H_ gene analysis in a series of 61 CLL cases, in order to investigate if the unique telomere lengthening shown in GC B cells could reflect the telomere status in the two subsets of mutated and unmutated CLL. A novel association was found between V_H_ gene mutation status and telomere length, since significantly shorter telomeres were demonstrated in the unmutated group compared to the mutated group (mean length 4.3 *vs* 6.3 kbp). Shorter telomeres also constituted a subgroup with a worse prognosis than cases with longer telomeres (median survival 59 *vs* 159 months). Furthermore, the Ig gene sequence data revealed that samples with high mutations frequency (>6%) had long telomeres (∼8 kbp). Thus, both the telomere and V_H_ gene mutation status in CLL appear linked, which may reflect the proliferative history of the clonal cells with regard to the GC reaction.

B-cell chronic lymphocytic leukaemia (CLL) is characterised by a monoclonal expansion of small B lymphocytes, typically expressing CD5, CD19, CD23 and low levels of surface immunoglobulin (Ig) (Matutes *et al*, 1994), which previously were supposed to originate from naive B cells of the mantle zone. However, recent analyses of the Ig heavy-chain variable region (IgV_H_) gene have defined two subsets of CLL, comprising cases with somatically unmutated or mutated V_H_ genes (Fais *et al*, 1998; Hamblin *et al*, 1999; Damle *et al*, 1999). The unmutated cases are considered to originate from pregerminal centre (GC) B cells and the mutated from post-GC B cells. A more favourable prognosis has been shown for CLL cases with somatically mutated V_H_ genes compared with unmutated cases (Hamblin *et al*, 1999; Damle *et al*, 1999; Maloum *et al*, 2000; Thunberg *et al*, 2001), indicating that CLL can be separated into at least two entities with a different clinical outcome.

Human telomeres in normal somatic cells consist of 6–12 kbp of TTAGGG-repeats that are eroded upon cell division because of the so-called ‘end-replication problem’ (Moyzis *et al*, 1988; Blackburn, 1991; Levy *et al*, 1992). A decrease in telomere length has been demonstrated with cell division *in vitro* and with cellular age *in vivo* for different cell types and tissues. At a critical telomere length during shortening, an ageing programme is activated in normal cells (Allsopp *et al*, 1992,1995a,1995b). Thus, the telomere length can predict and limit the number of divisions a cell can undergo. This telomere erosion, however, can be counteracted by the telomerase complex, which has a reverse transcriptase activity and can synthesise new telomeric repeats (Morin, 1989). Telomerase activity is linked to cells with an extended or infinite division potential, such as stem cells, germline cells and lymphocytes as well as permanent cell lines and tumor cells, while telomerase activity is absent from most normal somatic cells (Kim *et al*, 1994; Hiyama *et al*, 1995; Härle-Bachor and Boukamp, 1996; Norrback *et al*, 1996; Norrback and Roos, 1997). Recently, high levels of telomerase activity have been demonstrated in normal GC B cells in association with a unique telomere lengthening process not demonstrated in other cell types *in vivo* (Hu *et al*, 1997; Weng *et al*, 1997; Norrback *et al*, 2001).

Different levels of telomerase activity are expressed in the vast majority of leukaemias and malignant lymphomas (Norrback and Roos, 1997). In acute myeloid leukaemia, relapse cases demonstrate higher telomerase activity levels compared to that at diagnosis and similar findings have been reported for advanced CLL cases (Counter *et al*, 1995; Ohyashiki *et al*, 1997; Xu *et al*, 1998). These data indicate that the expression of telomerase activity can be associated with tumour progression in haematological neoplasms. Regarding CLL, one study has shown that both the telomere length and telomerase activity level were significant prognostic markers, since a short median survival was significantly associated with short telomere length (<6 kbp) and high telo-merase activity (Bechter *et al*, 1998). If the telomere lengthening in GC B cells demonstrated in benign tissues, like tonsils, is of relevance in this context, CLL cases of post-GC type should then be expected to have longer telomeres than pre-GC CLL.

In the present study, we have analysed a series of CLL cases for V_H_ mutation status by sequencing of the clonal rearrangements, for telomere length by Southern blotting and for clinical outcome. We have found an association between V_H_ mutation status and telomere length with shorter telomeres in unmutated (pre-GC) CLL cases compared with mutated (post-GC) CLL. Furthermore, a relationship between V_H_ mutation and telomere length was demonstrated since a gradual increase in telomere length was shown to parallel a decrease in homology to V_H_ germline sequences, that is, an increase in mutation rate.

## MATERIALS AND METHODS

### Patients

Frozen tumour samples from 61 patients were studied, which were identified in the archives of the departments of Pathology at Uppsala University Hospital and Umeå University Hospital between 1981 and 1998. The tumour material was obtained from bone marrow (29 cases), peripheral blood (26 cases), spleen (five cases) and lymph node (one case). There were 43 men and 18 women. Survival data were available for 56 patients from the local Swedish population and cancer registries in Uppsala and Umeå. The median age at diagnosis was 65 years and the median survival time was 82 months. Patient follow-up ranged from 8 to 182 months with a median follow-up of 56 months. The present study was approved by the ethical committee.

### Morphology and immunophenotyping

Classification was performed on smears, sections, imprints and by immunophenotyping using flow cytometry. According to the Royal Marsden scoring system the tumour cells expressed CD5, CD19 and CD23, and a weak expression of Ig (Matutes *et al*, 1994).

### PCR amplification and nucleotide sequencing analysis

High molecular weight DNA was prepared from frozen tumour material using standard protocols including proteinase K treatment, chloroform treatment and ethanol precipitation. V_H_ gene family-specific PCR amplification was performed using six family- specific V_H_ primers and one J_H_ primer as previously described (Li *et al*, 1999). Monoclonal PCR products were distinguished from polyclonal by a single-strand conformation polymorphism analysis using polyacrylamide gel or GenePhor electrophoresis according to the manufacturer's protocol (Amersham Pharmacia biotech, Uppsala, Sweden).

The clonal PCR products were sequenced directly using the BigDye Terminator Cycle Sequencing Reaction Kit (Perkin-Elmer, ABI, FosterCity, CA, USA) or using cloning as previously described (Li *et al*, 1999). All sequence reactions were analysed using an automated DNA sequencer (ABI 377, Applied Biosystems, Foster City, CA, USA).

### Analysis of V_H_, D and J_H_ sequences

The sequences were aligned to IgH sequences from the BLAST database (National Center for Biotechnology Information, USA), the V-BASE database (MRC, Centre for Protein Engineering, Cambridge, UK) and the Immunogenetics database (http://imgt.cines.fr:8104, initiator and coordinator: Marie-Paule Lefranc, Montpellier, France). A V_H_ gene sequence deviating more than 2% from the corresponding germline gene was defined as mutated (Matsuda *et al*, 1993).

### Southern blotting

Southern blotting and hybridisation with the telomeric probe (TTAGGG)_4_ were performed and mean telomere restriction fragment (TRF) length was calculated as previously described (Mehle *et al*, 1994). As molecular weight standards, the lambda DNA/*Eco*1 *Styl*/*Mlu*1 Marker (MBI Fermentas Inc., Amherst, NY, USA) and the DNA molecular weight marker X (Boehringer Mannheim Gmbh, Germany) were used. The peak TRF value was estimated as the length corresponding to the point with the highest optical density within the TRF profile.

### hTERT mRNA expression

In 19 cases with material available for RNA extraction, hTERT mRNA levels were analysed as previously described (Norrback *et al*, 2001) using a real-time polymerase chain reaction. The amount of hTERT mRNA was quantified using the Light Cycler Telo TAGGG hTERT Quantification kit where hTERT RNA levels were expressed as a ratio between the expression level of hTERT RNA and a house-keeping gene RNA (porphobilinogen deaminase) according to the manufacturer's protocol (Roche, Basel, Switzerland).

### Statistical analyses

Kaplan–Meier survival curves and the log-rank test were performed to study the prognostic significance of V_H_ gene mutations and telomere length in CLL using the Statistica 5.5 software (Stat Soft Inc., Tulsa, USA). Survival was calculated from the date of diagnosis until the last follow-up or death. Independent samples *t*-test was used to compare means, and nonparametric correlation was calculated according to the Spearman rank correlation. Probabilities of less than 0.05 were accepted as a significant value.

## RESULTS

### Analysis of V_H_ gene mutations

A total of 34 cases (56%) demonstrated unmutated V_H_ genes and 27 cases (44%) somatically mutated V_H_ genes. In the mutated cases, the mutation frequency ranged from 2.1–11.7%. Two clonal rearrangements were found in six cases, five of which showed two unmutated V_H_ genes, whereas one case had one unmutated and one mutated (97.7% homology) V_H_ gene. The last case was considered as mutated and the first five cases as unmutated.

### Telomere length and Ig gene mutation status

A summary of the data is given in Table 1

**Table 1:** Telomere length in relation to immunoglobulin V_H_ gene mutation status in a series of 61 CLL cases

	**Mean TRF (kbp)**			**Peak TRF (kbp)**		
**V_H_ gene status**	**Mean**	**Median**	**Range**	**Mean**	**Median**	**Range**
Unmutated (*n*=34)	4.29	4.24	3.06–5.62	4.29	4.24	2.35–7.66
Mutated (*n*=27)	5.36	5.07	3.30–7.36	6.25	5.69	2.66–10.10
All cases (*n*=61)	4.76	4.49	3.06–7.36	5.16	4.57	2.35–10.10

. The mean peak TRF length was 5.16 kbp and the median peak TRF length was 4.57 kbp, range 2.35–10.10 kbp. The reason for using peak TRF values in the further analysis was that 10 cases had two peaks and for these the value of the largest peak was used, since all cases had >50% monoclonal B cells as determined by immunophenotyping. The cases with double peaks had only one clonal Ig gene rearrangement.

**Figure 1:**
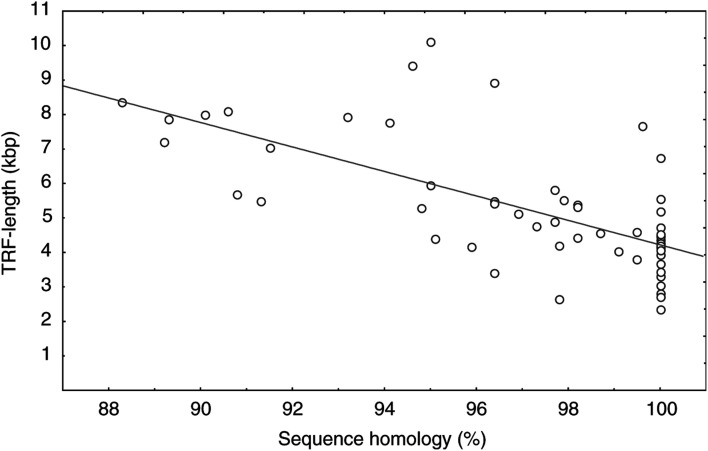
Correlation between telomere length in kbp and V_H_ gene mutation frequency given as percent homology with germline (*r*^2^=0.46, *P*<0.001).

A total of 34 unmutated and 27 mutated samples were analysed for telomere length. The unmutated cases demonstrated a median peak TRF length of 4.24 kbp (mean peak 4.29 kbp, range 2.35–7.66 kbp) compared with 5.69 kbp (mean peak 6.25 kbp, range 2.66–10.10 kbp) for the mutated cases. The association between mutation status and telomere length (Figure 1

) was significant using the Spearman rank correlation (*R*^2^=0.46, *P*<0.001).

Thus, the IgV_H_ gene mutation status correlated with TRF length. For cases with no somatic hypermutations (i.e. 100% homology to the germline gene), the TRF length was ∼4 kpb and for cases with a high mutation rate (<94% homology to the germline gene), the TRF length was ∼8 kbp. The material was divided into two groups of equal size with a cutoff at the median peak TRF value (4.57 kbp).

The presence of hTERT RNA was detected in all the 19 cases studied. Using independent samples' *t*-test, a significant association (*P*=0.032) was found between long telomeres (TRF >4.57 kbp) and low hTERT mRNA levels and *vice versa* (data not shown). No association was found between mutation status and hTERT mRNA levels.

### Survival analysis

**Figure 2:**
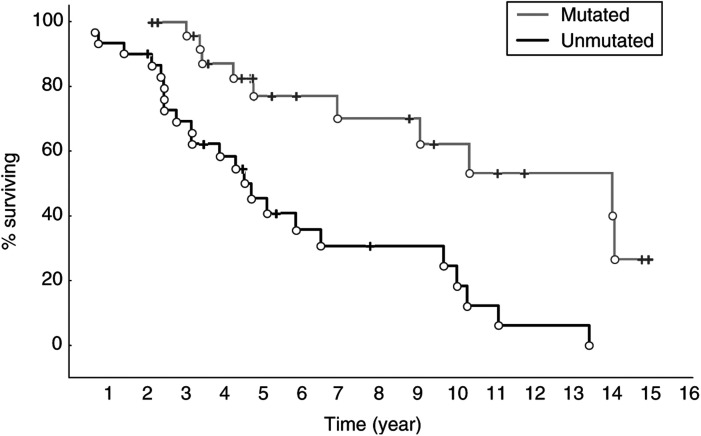
Survival data for V_H_ gene mutated (26 patients) and unmutated (30 patients) cases demonstrating a significant difference in overall survival (*P*<0.001).

Survival curves were plotted according to the Kaplan–Meier method using data available on 56 patients. The survival data were grouped into mutated (26 patients) and unmutated (30 patients) cases and a statistically significant difference in overall survival was found between these two groups (*P*<0.001) (Figure 2

). The median survival for patients with mutated V_H_ genes was 138 months and for those without mutations 55 months, calculated from the date of diagnosis. The median age was 63 years for patients with unmutated V_H_ genes and 67 years for patients with mutated V_H_ genes.

**Figure 3:**
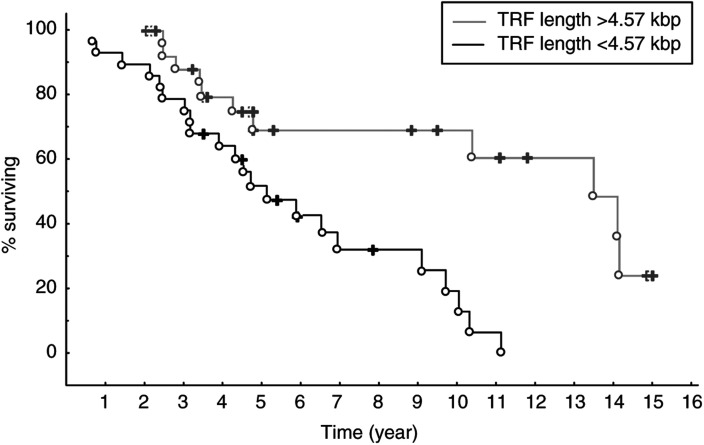
Survival data for CLL patients subdivided according to telomere length with a cutoff at the median peak TRF value of 4.57 kbp. A significant difference in survival was found between the two groups with a better survival for cases with TRF values >4.57 kbp (*P*=0.0015).

Regarding telomere length, survival analysis was performed after dividing the material into two groups with a cutoff at the median peak TRF value (4.57 kbp). A significant difference in survival was found between the two groups with a better overall survival for cases with peak TRF values >4.57 kbp (*P*=0.0015) (Figure 3

). The median survival for patients with telomeres longer than the cutoff value was 159 months and for those with shorter telomeres 59 months. The median age was 62 years for patients with TRF lengths <4.57 kbp and 67 years for cases with TRF lengths >4.57 kbp. Using Cox regression analysis neither mutation status nor telomere length were independent variables, indicating an association between these two parameters.

## DISCUSSION

Convincing data have made it probable that CLL consists of at least two variants derived from different subpopulations of neoplastic cells (Fais *et al*, 1998; Damle *et al*, 1999; Hamblin *et al*, 1999; Maloum *et al*, 2000), one originating from unmutated pre-GC cells and the other from somatically hypermutated GC or post-GC cells. This notion was further supported by the observation that patients with mutated V_H_ genes had a significantly better outcome compared to patients lacking V_H_ gene mutations (Damle *et al*, 1999; Hamblin *et al*, 1999; Maloum *et al*, 2000; Thunberg *et al*, 2001). In the present study, we were able to verify these data regarding prognosis and V_H_ gene mutation status, since our mutated CLL cases displayed more than twice as long median survival than the unmutated cases (138 *vs* 55 months). Most interestingly, we found a novel association between V_H_ gene mutation frequency and telomere length, showing significantly longer telomeres in CLL cases having somatically mutated V_H_ genes. We could also demonstrate that CLL cases with longer telomeres had a significantly better prognosis than cases with shorter telomeres (159 *vs* 59 months), supporting a previously published study (Bechter *et al*, 1998). But what could be the biological explanation for this new association coupling together our findings?

Normal lymphocytes have been shown to lose telomeres with each cell division *in vivo* and *in vitro* (Vaziri *et al*, 1994; Weng *et al*, 1995; Norrback and Roos, 1997; Rufer *et al*, 1998). However, GC B cells are characterised by telomerase activity and a unique telomere lengthening process has been demonstrated in crude preparations of GC B cells (Hu *et al*, 1997; Weng *et al*, 1997; Norrback *et al*, 2001). In immunopurified cell populations from tonsils, we found a telomere lengthening in both the centroblast and centrocyte populations (Norrback *et al*, 2001). The telomere length increase is likely to occur in proliferating pre-GC blasts and centroblasts, since telomerase activity is coupled to cell cycle progression in lymphocytes (Buchkovich and Greider, 1996; Igarashi and Sakaguchi, 1997). In cells maturing from naive B cells to centroblasts/centrocytes, we could therefore demonstrate a telomere lengthening of up to 4 kbp in individual cases (Norrback *et al*, 2001).

In our CLL material, mutated cases showed roughly 2 kb longer telomeres than the unmutated. We therefore believe that this difference in telomere length between mutated and unmutated CLL reflects, and supports the theory, that these subsets probably originate from different stages in the B-cell development, that is, pre- and post-GC B cells. Interestingly, the difference in telomere length between pre-GC and post-GC cells seemed to be preserved in the CLL cases, which may indicate a similar rate of telomere loss in the different CLL clones. The shorter telomere lengths in unmutated and mutated CLL compared to normal pre-GC and GC B cells (Norrback *et al*, 2001) could, at least partly, be explained by telomere loss normally occurring during ageing, since most of the normal GC B cells studied by us were derived from tonsils from young individuals whereas the CLL cases represented an older patient group.

Telomerase is activated in the vast majority of leukaemias and malignant lymphomas, including CLL (reviewed in Norrback and Roos, 1997). In a subset of our CLL cohort (*n*=19), we have analysed the mRNA levels of the catalytic unit of telomerase, hTERT, which have been shown to correlate with the telomerase activity in B lymphocytes (Liu *et al*, 1999; Norrback *et al*, 2001). We found that CLL cases with longer telomeres had lower hTERT mRNA levels than cases with shorter telomeres, which is in line with a previously published report on telomerase activity (Bechter *et al*, 1998). These collected data argue that the difference in telomere lengths between the two groups seemed unrelated to hTERT RNA levels (and thus telomerase activity), and rather reflected the telomere status of the primarily transformed cells.

One common denominator for telomerase activation and the V_H_ gene hypermutation mechanism in the GC is an active cell cycle. Somatic V_H_ gene hypermutations occur in growing B cells given the survival signals based on antigen selection, and in these cells telomerase is known to be upregulated as discussed above. The combined telomere length and V_H_ gene hypermutation analysis presented here might give new insights into the kinetics of the hypermutation machinery. It can be anticipated that the total telomere length increase is a result of the number of cell cycle rounds undergone in the GC. Thus, the data suggest that a limited number of V_H_ gene mutations occur for each GC cell division round since post-GC B cells with long telomeres had high V_H_ gene mutation frequencies. This association between V_H_ gene mutation status and telomere length provides new possibilities for more detailed analyses of GC B cell subpopulations. It should be noted that an alternative way to acquire somatic V_H_ gene mutations has been described in the hyper-IgM syndrome because of a nonfunctional CD40–CD154 interaction leading to no development of germinal centres (Durandy and Honjo, 2001). However, the mutation frequency in the hyper-IgM syndrome (usually <2%) (Chu *et al*, 1995; Monson *et al*, 2001; Weller *et al*, 2001) is below the cutoff level used to distinguish between mutated and unmutated CLL cases. Hence, it seems unlikely that the mutated CLL cases had acquired V_H_ gene mutations by the alternative CD40-independent pathway.

Finally, the survival data also suggest that telomere length can be an important clinical parameter to study for the evaluation of CLL in the diagnostic setting, since longer telomere lengths were associated with better clinical outcome. We have chosen the median value as cutoff level, but the true border to divide the material into prognostic groups deserves further investigation. Recently described techniques for flow cytometric analysis after *in situ* hybridisation with a fluorochrome labelled telomere probe (‘flow-FISH’) can be useful (Rufer *et al*, 1998; Hultdin *et al*, 1998), since the flow-FISH approach is convenient and can be directly applied to material sent for regular diagnostic work. However, further studies of larger groups of patients have to be performed to clarify the clinical impact of telomere length analysis in CLL.
